# Supplementary material for: Lipid-Based Nanocarriers for Neurological Disorders: A Review of the State-of-the-Art and Therapeutic Success to Date
Source: Pharmaceutics. 2022 Apr 11;14(4):836. doi: 10.3390/pharmaceutics14040836 (PMC9031624; doi:10.3390/pharmaceutics14040836)
Supplement: Supplementary file 1 [file pharmaceutics-14-00836-s001.zip › pharmaceutics-1631478-supplementary.pdf]

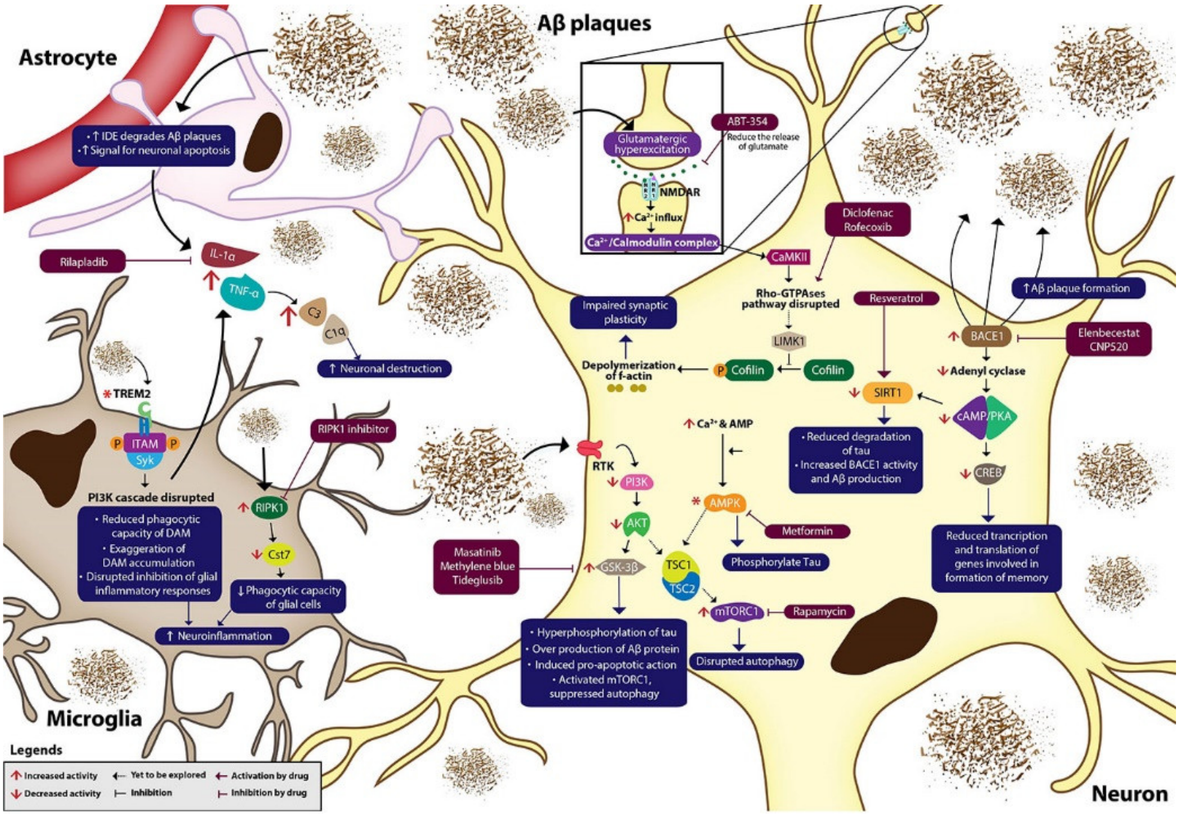

Figure S1. Mechanisms of neuroinflammation in Alzheimer's disease and drug targets [Obtained and reproduced without any changes from [1] and Frontiers in Pharmacology in accordance with **Creative Commons Attribution License**] **(CC BY)**].

Key: IDE, insulin-degrading enzyme; Aβ, amyloid βeta; IL-1α, interleukin 1α; TNF-α, tumour necrosis factor-α; C3, complement component 3; C1q, complement protein 1q; TREM2, triggering receptor expressed on myeloid cells 2; ITAM, immunoreceptor tyrosine-based activation motif; SYK, spleen tyrosine kinase; P, phosphate; PI3K, phosphatidylinositol 3-kinase; NFκB, nuclear factor kappa β; RIPK1, receptor-interacting serine/threonine-protein kinase 1; Cst7, cystatin F gene; RTK, receptor tyrosine kinase; PDK1, phosphoinositide-dependent kinase 1; mTOR, mammalian Target of Rapamycin; Akt, protein kinase B; GSK-3β, glycogen synthase kinase 3β; TSC, tuberous sclerosis complex; AMP, adenosine monophosphate; AMPK, AMP-activated protein kinase; SIRT1, silent information regulator type 1; BACE1, β-secretase 1; cAMP, cyclic adenosine monophosphate; PKA, protein kinase A; CREB, cAMP response element binding protein; NMDAR, NMDA receptor; Ca^{2+}/Calmodulin-dependent protein kinase II (CAMKII); LIMK1, LIM kinase 1; BDNF, brain-derived neurotrophic factor. Asterisk (*) in the diagram indicates uncertain changes of activity in AD.

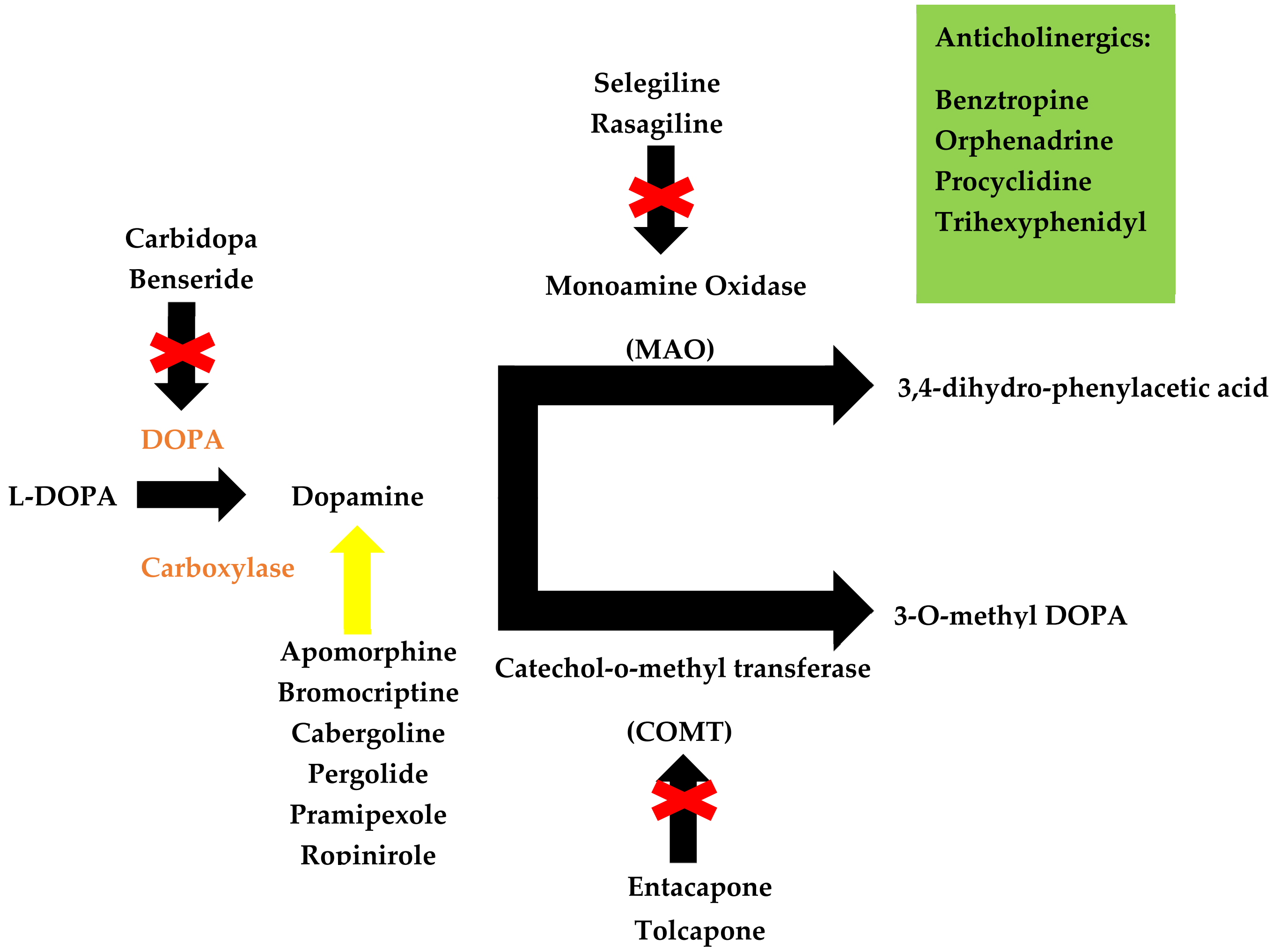

Figure S2. Summary of the mechanisms of action of the drugs used to treat Parkinson's Disease.

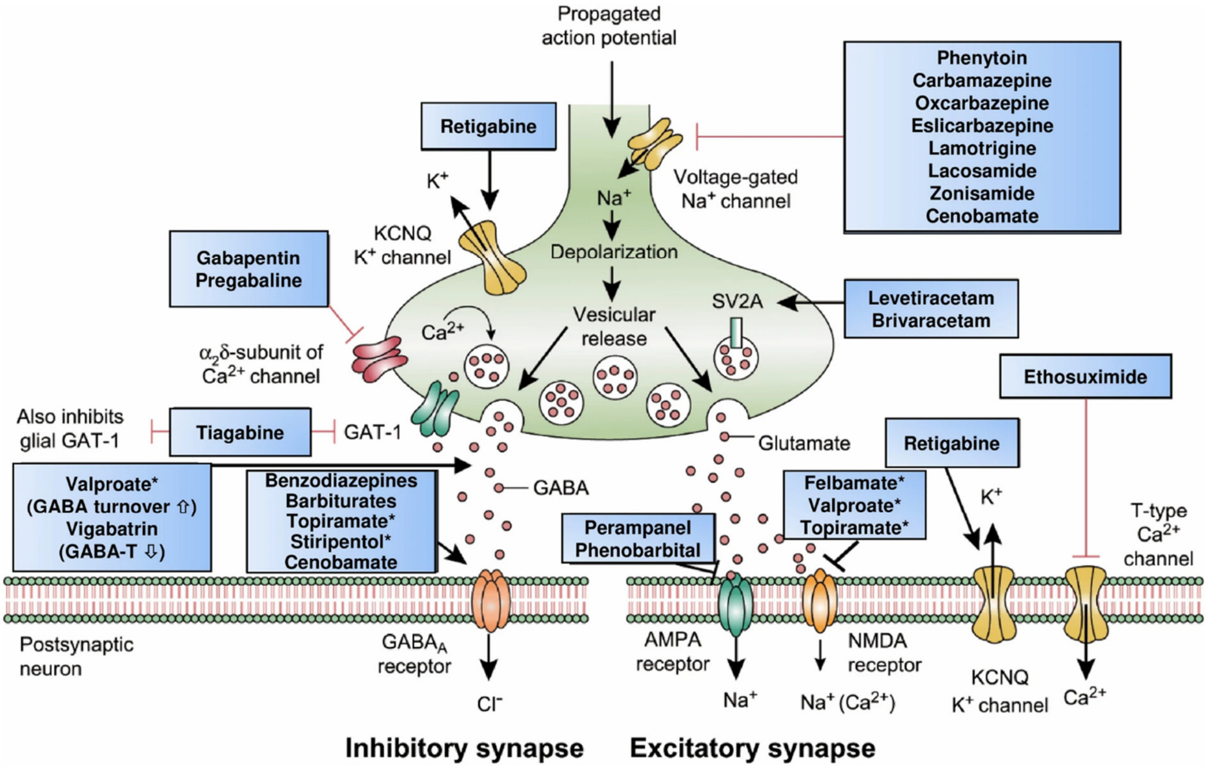

Figure S3. Mechanism of action of clinically approved antiepileptic drugs (AAED). [Obtained and reproduced without any changes from [2] and Springer in accordance with **Creative Commons Attribution License (CC BY)**]. The asterisks are used to indicate that the compounds elicit their action through multiple mechanisms of which not all are depicted.

Key: AMPA-α-amino-3-hydroxy-5-methyl-4-isoxazolepropionic acid, GABA-γ-aminobutyric acid, GABA-T- GABA aminotransferase, GAT-1 GABA transporter 1, KCNQ Kv7 potassium channel family, NMDA N-methyl-D-aspartate, SV2A synaptic vesicle protein 2A

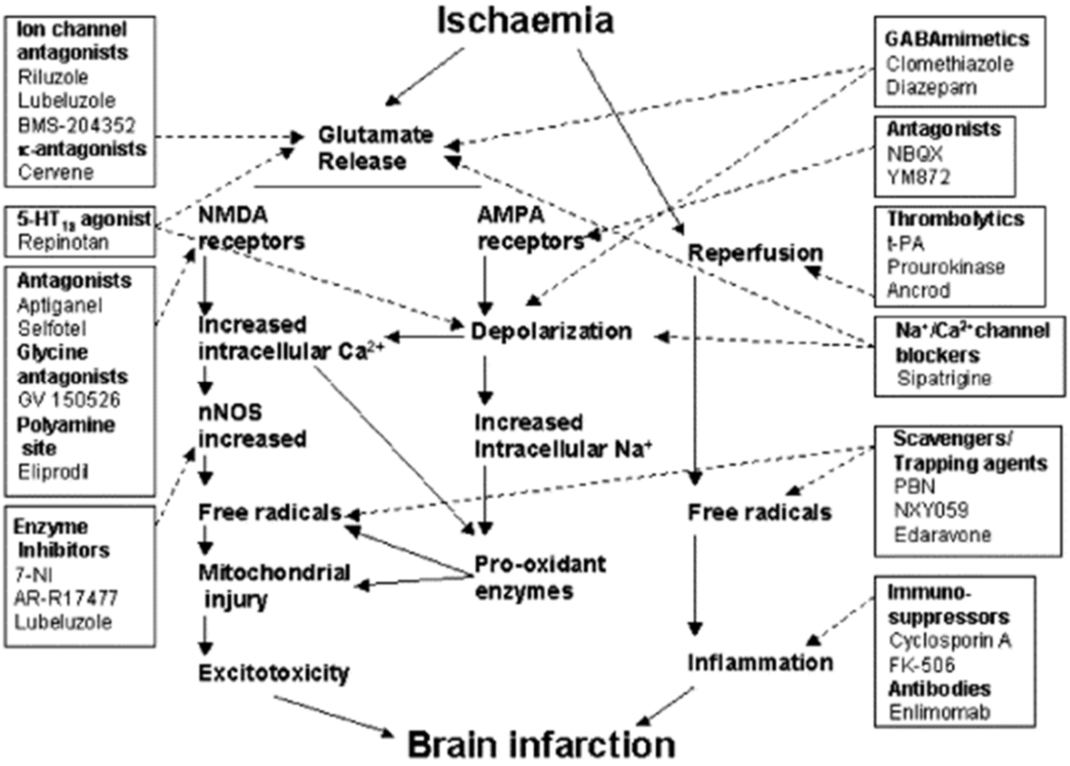

Figure S4. Schematic representation of the ischemic cascade showing compounds developed to interfere with mechanisms and provide neuroprotection. Reproduced from [3] with permission from Elsevier.
